# *catena*-Poly[{μ_3_-4,4′,6,6′-tetra­chloro-2,2′-[butane-1,4-diylbis(nitrilo­methanyl­yl­idene)]diphenolato}{μ_2_-4,4′,6,6′-tetra­chloro-2,2′-[butane-1,4-diylbis(nitrilo­methanylyl­idene)]diphenolato}dicopper(II)]

**DOI:** 10.1107/S1600536812028462

**Published:** 2012-06-30

**Authors:** Reza Kia, Hadi Kargar, Amir Adabi Ardakani, Muhammad Nawaz Tahir

**Affiliations:** a Department of Chemistry, Science and Research Branch, Islamic Azad University, Tehran, Iran; b Structural Dynamics of (Bio)Chemical Systems, Max Planck Institute for Biophysical Chemistry, Am Fassberg 11, 37077 Göttingen, Germany; c Department of Chemistry, Payame Noor University, PO Box 19395-3697 Tehran, I. R. of IRAN; d Department of Physics, University of Sargodha, Punjab, Pakistan

## Abstract

The asymmetric unit of the title compound, [Cu_2_(C_18_H_14_Cl_4_N_2_O_2_)_2_]_*n*_, contains two independent Cu^II^ ions which are bridged by a pair of 4,4′,6,6′-tetra­chloro-2,2′-[butane-1,4-diylbis(nitrilo­methanylyl­idene)]diphenolate ligands, forming a dinuclear unit. One of the Cu^II^ ions is coordinated in a distorted square-planar environment and the other is coordinated in a distorted square-pyramidal environment. The long apical Cu—O bond of the square-pyramidal coordinated Cu^II^ ion is formed by a symmetry-related O atom, creating a one-dimensional polymer along [010]. In addition, short inter­molecular Cl⋯Cl distances [3.444 (2) Å] and weak π–π inter­actions [centroid–centroid distances = 3.736 (2)–3.875 (3) Å] are observed. The crystal studied was an inversion twin with a refined twin component ratio of 0.60 (1):0.40 (1).

## Related literature

For van der Waals radii, see: Bondi (1964 ▶). For background to coordination polymers, see: Kido & Okamoto (2002 ▶); Li *et al.* (2006 ▶). For bis-bidentate Schiff base complexes, see: Hannon *et al.* (1999 ▶); Lavalette *et al.* (2003 ▶). For the synthesis and structural variations of Schiff base complexes, see: Granovski *et al.* (1993 ▶); Elmali *et al.* (2000 ▶). For related structures, see: Kargar & Kia (2011**a* ▶,b* ▶).
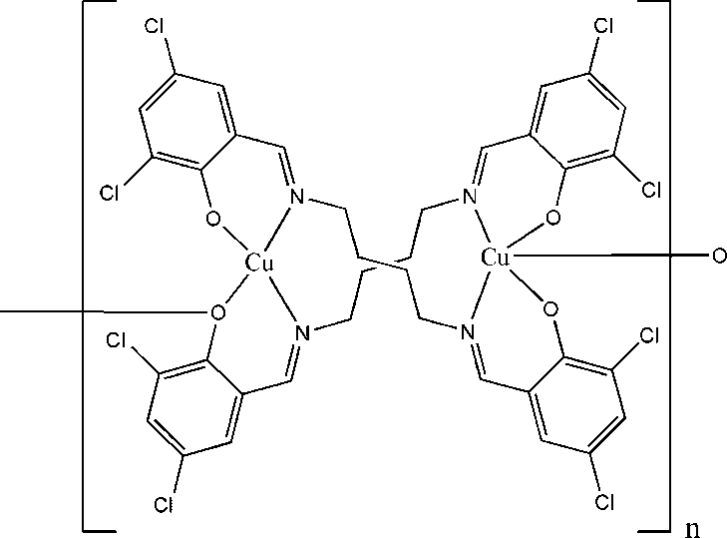


## Experimental

### 

#### Crystal data

[Cu_2_(C_18_H_14_Cl_4_N_2_O_2_)_2_]
*M* *_r_* = 990.30
Orthorhombic, 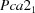

*a* = 26.6927 (16) Å
*b* = 7.7775 (4) Å
*c* = 18.6689 (9) Å
*V* = 3875.7 (4) Å^3^
*Z* = 4
Mo *K*α radiation
μ = 1.70 mm^−1^
*T* = 291 K
0.36 × 0.18 × 0.16 mm

#### Data collection

Bruker SMART APEXII CCD diffractometer
Absorption correction: multi-scan (*SADABS*; Bruker, 2001 ▶) *T*_min_ = 0.581, *T*_max_ = 0.773
18623 measured reflections
8990 independent reflections
6793 reflections with *I* > 2σ(*I*)
*R*_int_ = 0.034

#### Refinement

*R*[*F* ^2^ > 2σ(*F* ^2^)] = 0.041
*wR*(*F* ^2^) = 0.085
*S* = 0.99
8990 reflections
488 parameters
1 restraint
H-atom parameters constrained
Δρ_max_ = 0.43 e Å^−3^
Δρ_min_ = −0.35 e Å^−3^
Absolute structure: Flack (1983 ▶), 4247 Friedel pairs
Flack parameter: 0.605 (10)

### 

Data collection: *APEX2* (Bruker, 2007 ▶); cell refinement: *SAINT* (Bruker, 2007 ▶); data reduction: *SAINT*; program(s) used to solve structure: *SHELXS97* (Sheldrick, 2008 ▶); program(s) used to refine structure: *SHELXL97* (Sheldrick, 2008 ▶); molecular graphics: *SHELXTL* (Sheldrick, 2008 ▶); software used to prepare material for publication: *SHELXTL* and *PLATON* (Spek, 2009 ▶).

## Supplementary Material

Crystal structure: contains datablock(s) global, I. DOI: 10.1107/S1600536812028462/lh5494sup1.cif

Structure factors: contains datablock(s) I. DOI: 10.1107/S1600536812028462/lh5494Isup2.hkl

Additional supplementary materials:  crystallographic information; 3D view; checkCIF report

## Figures and Tables

**Fig. 1.**
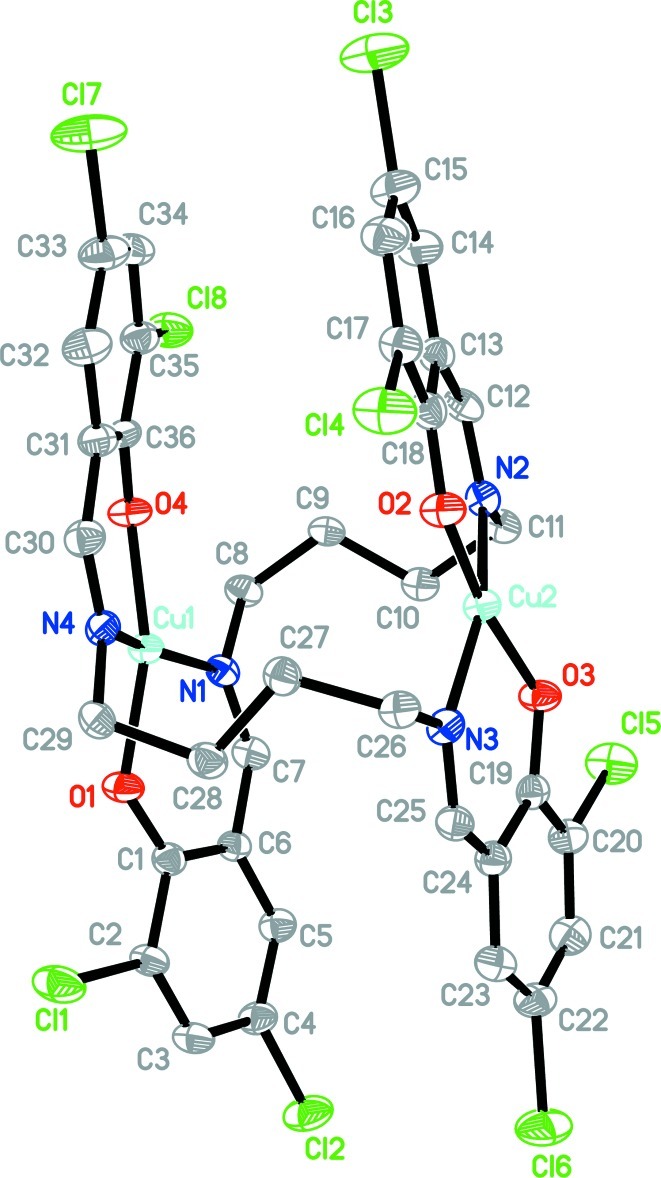
The asymmetric unit of the title complex, showing 40% probability displacement ellipsoids [H-atoms have been omitted for clarity].

**Fig. 2.**
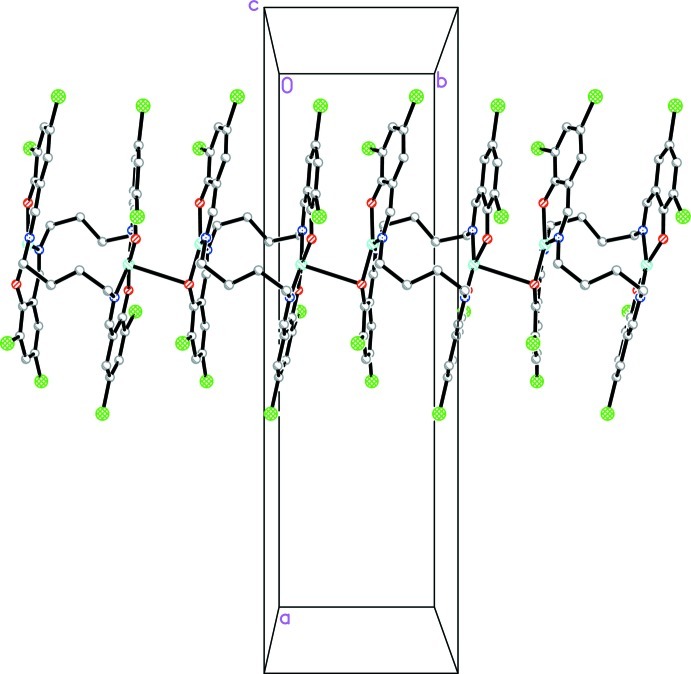
Part of the crystal structure, viewed along the c-axis, showing the one-dimensional coordination chain propagating along the b-axis [H-atoms have been omitted for clarity].

**Fig. 3.**
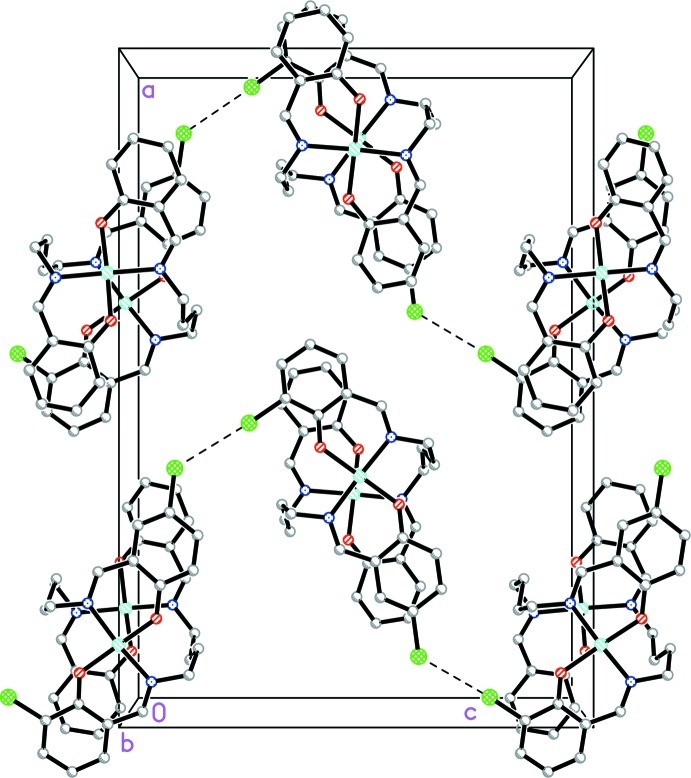
Part of the crystal structure, viewed along the b-axis, showing zig-zag linking of molecules through intermolecular Cl···Cl interactions along the c-axis [only the Cl atoms involved in the interactions are shown and H-atoms omitted for clarity].

## supplementary crystallographic information

### Comment

The design and construction of metal-organic coordination polymers (MOCPs) have attracted considerable attention, not only for their novel topologies but also for their potential in the area of magnetic applications and functional materials (Kido & Okamoto, 2002; Li *et al.*, 2006). One of the key strategies in the construction of metal-organic coordination polymers is to select suitable bi- or multi-dentate bridging ligands. Among these, bis-bidentate *NN*- or *NO*-donor Schiff base ligands with aliphatic and aromatic spacers (Hannon *et al.*, 1999; Lavalette *et al.*, 2003) have attracted much attention because of the flexibility in their coordination modes and the resulting intermolecular interactions. The long chain aliphatic spacers or rigid aromatic spacers with large bite angles in these ligands favour the bis-bidentate coordination mode and allow the ligands to accomodate metal centers in one unit of the ligand. On the other hand, Schiff bases are one of the most prevalent ligands in coordination chemistry and their complexes are some of the most important stereochemical models in transition metal-organic chemistry, with their ease of preparation and structural variations (Granovski *et al.*, 1993; Elmali *et al.*, 2000).

The asymmetric unit of the title complex is shown in Fig. 1. The bond lengths and angles are comparable to those in related structures (Kargar & Kia, 2011**a*,b*). The long apical Cu—O bond is shorter than the sum of the van der Waals (vdW) radii of these atoms [Cu, 1.43Å and O, 1.52 Å; Bondi, 1964] and is formed by a symmetry related O atom creating a one-dimenional polymer along [010] (Fig. 2). In the crystal there are intermolecular Cl4···Cl6(1/2 - x, y, -1/2 + z) [3.444 (2)Å] distances which are shorter than the sum of the van der Waals radii for Cl [3.50Å] atoms (Bondi, 1964; Fig. 3). In addition, intermolecular π–π interactions [*Cg*1···*Cg*2^i^ = 3.736 (2)Å, (i) x, -1+y, z; *Cg*3···*Cg*4^ii^ = 3.875 (3) Å, (ii) x, 1+y, z; *Cg*1,*Cg*2, *Cg*3 and *Cg*4 are the centroids of the (C1–C6), (C6–C8), (C13–C18) and (C31–C36) rings respectively].

### Experimental

The title complex was synthesized by an methanolic solution (50 ml) of bis(3,5-chlorosalicylaldeyde)-1,4-butanediimine (2 mmol) and CuCl_2_.4H_2_O (2 mmol). After stirring at reflux conditions for 2 h, the solution was filtered and the resulting dark-red powder was crystallized from DMF, giving single crystals suitable for X-ray diffraction.

### Refinement

All H atoms were positioned geometrically and constrained to refine with the parents atoms using the riding-model approximation, with C—H = 0.93 - 0.97Å and *U*_iso_(H) = 1.2 *U*_eq_(C). The crystal used was an inversion twin with refined twin components ratio of 0.60 (1)/0.40 (1).

### Crystal data

**Table:**

[Cu_2_(C_18_H_14_Cl_4_N_2_O_2_)_2_]	*Z* = 4
*M**_r_* = 990.30	*F*(000) = 1988
Orthorhombic, *P**c**a*2_1_	*D*_x_ = 1.697 Mg m^−^^3^
Hall symbol: P 2c -2ac	Mo *K*α radiation, λ = 0.71073 Å
*a* = 26.6927 (16) Å	µ = 1.70 mm^−^^1^
*b* = 7.7775 (4) Å	*T* = 291 K
*c* = 18.6689 (9) Å	Needle, dark-red
*V* = 3875.7 (4) Å^3^	0.36 × 0.18 × 0.16 mm

### Data collection

**Table:**

Bruker SMART APEXII CCD diffractometer	8990 independent reflections
Radiation source: fine-focus sealed tube	6793 reflections with *I* > 2σ(*I*)
Graphite monochromator	*R*_int_ = 0.034
φ and ω scans	θ_max_ = 27.9°, θ_min_ = 1.5°
Absorption correction: multi-scan (*SADABS*; Bruker, 2001)	*h* = −35→34
*T*_min_ = 0.581, *T*_max_ = 0.773	*k* = −10→10
18623 measured reflections	*l* = −24→23

### Refinement

**Table:**

Refinement on *F*^2^	Secondary atom site location: difference Fourier map
Least-squares matrix: full	Hydrogen site location: inferred from neighbouring sites
*R*[*F*^2^ > 2σ(*F*^2^)] = 0.041	H-atom parameters constrained
*wR*(*F*^2^) = 0.085	*w* = 1/[σ^2^(*F*_o_^2^) + (0.0355*P*)^2^] where *P* = (*F*_o_^2^ + 2*F*_c_^2^)/3
*S* = 0.99	(Δ/σ)_max_ = 0.001
8990 reflections	Δρ_max_ = 0.43 e Å^−^^3^
488 parameters	Δρ_min_ = −0.35 e Å^−^^3^
1 restraint	Absolute structure: Flack (1983), 4247 Friedel pairs
Primary atom site location: structure-invariant direct methods	Flack parameter: 0.605 (10)

### Special details

**Table:**

Geometry. All esds (except the esd in the dihedral angle between two l.s. planes) are estimated using the full covariance matrix. The cell esds are taken into account individually in the estimation of esds in distances, angles and torsion angles; correlations between esds in cell parameters are only used when they are defined by crystal symmetry. An approximate (isotropic) treatment of cell esds is used for estimating esds involving l.s. planes.
Refinement. Refinement of F^2^ against ALL reflections. The weighted R-factor wR and goodness of fit S are based on F^2^, conventional R-factors R are based on F, with F set to zero for negative F^2^. The threshold expression of F^2^ > 2sigma(F^2^) is used only for calculating R-factors(gt) etc. and is not relevant to the choice of reflections for refinement. R-factors based on F^2^ are statistically about twice as large as those based on F, and R- factors based on ALL data will be even larger.

### Fractional atomic coordinates and isotropic or equivalent isotropic displacement parameters (Å^2^)

**Table:**

	*x*	*y*	*z*	*U*_iso_*/*U*_eq_	
Cu1	0.337328 (15)	0.58203 (6)	0.49568 (2)	0.03303 (11)	
Cu2	0.372623 (16)	1.16823 (6)	0.50686 (2)	0.03338 (11)	
Cl1	0.16981 (4)	0.56157 (19)	0.41600 (6)	0.0637 (4)	
Cl2	0.10357 (4)	0.81832 (18)	0.66592 (6)	0.0630 (3)	
Cl3	0.62687 (5)	0.9809 (2)	0.39111 (8)	0.0819 (5)	
Cl4	0.44901 (4)	1.14708 (19)	0.27711 (5)	0.0632 (4)	
Cl5	0.30179 (5)	1.27744 (18)	0.73675 (6)	0.0610 (3)	
Cl6	0.11528 (5)	1.28198 (19)	0.62737 (8)	0.0720 (4)	
Cl7	0.57210 (5)	0.5842 (2)	0.30261 (8)	0.0873 (5)	
Cl8	0.50484 (4)	0.49330 (18)	0.57186 (6)	0.0551 (3)	
O1	0.26712 (9)	0.5760 (4)	0.48373 (13)	0.0397 (7)	
O2	0.41409 (10)	1.1451 (4)	0.42485 (13)	0.0409 (7)	
O3	0.33216 (9)	1.2300 (4)	0.58740 (14)	0.0403 (7)	
O4	0.40552 (9)	0.5174 (3)	0.50582 (15)	0.0408 (6)	
N1	0.33065 (11)	0.6318 (4)	0.60038 (16)	0.0339 (8)	
N2	0.42848 (12)	1.1052 (4)	0.57284 (16)	0.0334 (7)	
N3	0.31196 (12)	1.1706 (4)	0.44287 (17)	0.0357 (8)	
N4	0.34356 (12)	0.6048 (4)	0.38931 (17)	0.0338 (8)	
C1	0.23262 (14)	0.6340 (5)	0.5258 (2)	0.0345 (9)	
C2	0.18203 (13)	0.6377 (5)	0.5021 (2)	0.0386 (8)	
C3	0.14355 (15)	0.6947 (6)	0.5433 (2)	0.0435 (10)	
H3	0.1111	0.6963	0.5253	0.052*	
C4	0.15322 (15)	0.7505 (6)	0.6124 (2)	0.0411 (10)	
C5	0.20089 (14)	0.7489 (5)	0.6389 (2)	0.0389 (9)	
H5	0.2070	0.7873	0.6853	0.047*	
C6	0.24090 (14)	0.6895 (5)	0.5963 (2)	0.0326 (8)	
C7	0.28933 (14)	0.6746 (5)	0.6297 (2)	0.0370 (9)	
H7	0.2906	0.6990	0.6785	0.044*	
C8	0.37456 (14)	0.6148 (6)	0.6481 (2)	0.0368 (10)	
H8A	0.3928	0.5111	0.6357	0.044*	
H8B	0.3632	0.6036	0.6972	0.044*	
C9	0.40930 (13)	0.7679 (5)	0.6423 (2)	0.0364 (9)	
H9A	0.4388	0.7461	0.6713	0.044*	
H9B	0.4202	0.7788	0.5930	0.044*	
C10	0.38622 (14)	0.9362 (5)	0.6657 (2)	0.0368 (9)	
H10A	0.3795	0.9313	0.7167	0.044*	
H10B	0.3544	0.9511	0.6413	0.044*	
C11	0.41906 (15)	1.0905 (5)	0.65037 (19)	0.0371 (9)	
H11A	0.4027	1.1940	0.6675	0.045*	
H11B	0.4506	1.0785	0.6756	0.045*	
C12	0.47348 (15)	1.0699 (6)	0.5522 (2)	0.0369 (10)	
H12	0.4964	1.0428	0.5880	0.044*	
C13	0.49213 (14)	1.0681 (5)	0.47969 (19)	0.0357 (9)	
C14	0.54318 (16)	1.0277 (6)	0.4704 (2)	0.0485 (12)	
H14	0.5629	1.0001	0.5099	0.058*	
C15	0.56376 (17)	1.0291 (6)	0.4032 (2)	0.0527 (12)	
C16	0.53470 (17)	1.0663 (6)	0.3442 (2)	0.0484 (11)	
H16	0.5490	1.0660	0.2988	0.058*	
C17	0.48514 (15)	1.1036 (6)	0.3521 (2)	0.0406 (10)	
C18	0.46083 (16)	1.1077 (5)	0.4208 (2)	0.0377 (10)	
C19	0.28383 (14)	1.2284 (5)	0.5951 (2)	0.0339 (9)	
C20	0.26211 (15)	1.2550 (6)	0.6637 (2)	0.0410 (10)	
C21	0.21145 (17)	1.2690 (6)	0.6737 (2)	0.0476 (11)	
H21	0.1986	1.2889	0.7193	0.057*	
C22	0.17914 (16)	1.2535 (6)	0.6154 (2)	0.0472 (11)	
C23	0.19810 (15)	1.2208 (6)	0.5491 (2)	0.0445 (11)	
H23	0.1764	1.2078	0.5105	0.053*	
C24	0.24970 (15)	1.2066 (5)	0.5385 (2)	0.0381 (10)	
C25	0.26634 (15)	1.1856 (5)	0.4653 (2)	0.0378 (10)	
H25	0.2415	1.1826	0.4304	0.045*	
C26	0.31740 (15)	1.1496 (5)	0.36388 (19)	0.0365 (9)	
H26A	0.3446	1.2217	0.3471	0.044*	
H26B	0.2869	1.1879	0.3406	0.044*	
C27	0.32770 (15)	0.9657 (5)	0.3430 (2)	0.0374 (10)	
H27A	0.3576	0.9276	0.3679	0.045*	
H27B	0.3348	0.9623	0.2920	0.045*	
C28	0.28539 (14)	0.8383 (5)	0.3588 (2)	0.0396 (10)	
H28A	0.2754	0.8507	0.4085	0.048*	
H28B	0.2567	0.8669	0.3293	0.048*	
C29	0.29957 (15)	0.6540 (5)	0.3455 (2)	0.0371 (10)	
H29	0.2830	0.5809	0.3140	0.045*	
C30	0.38488 (16)	0.5911 (6)	0.3557 (2)	0.0408 (10)	
H30	0.3831	0.5974	0.3060	0.049*	
C31	0.43377 (14)	0.5672 (6)	0.3857 (2)	0.0397 (10)	
C32	0.47492 (17)	0.5804 (6)	0.3381 (2)	0.0497 (11)	
H32	0.4693	0.5975	0.2895	0.060*	
C33	0.52243 (17)	0.5682 (6)	0.3633 (3)	0.0536 (12)	
C34	0.53194 (16)	0.5446 (6)	0.4350 (2)	0.0489 (11)	
H34	0.5648	0.5399	0.4516	0.059*	
C35	0.49241 (14)	0.5279 (5)	0.4820 (2)	0.0403 (10)	
C36	0.44190 (14)	0.5369 (5)	0.4595 (2)	0.0365 (9)	

### Atomic displacement parameters (Å^2^)

**Table:**

	*U*^11^	*U*^22^	*U*^33^	*U*^12^	*U*^13^	*U*^23^
Cu1	0.0270 (2)	0.0393 (2)	0.0328 (2)	0.0001 (2)	0.0010 (2)	0.0004 (2)
Cu2	0.0324 (2)	0.0408 (3)	0.0269 (2)	0.0028 (2)	−0.0005 (2)	−0.0008 (2)
Cl1	0.0383 (6)	0.1084 (11)	0.0443 (6)	−0.0003 (7)	−0.0076 (5)	−0.0216 (7)
Cl2	0.0434 (6)	0.0912 (10)	0.0544 (7)	0.0174 (6)	0.0098 (5)	−0.0116 (7)
Cl3	0.0438 (7)	0.1340 (15)	0.0678 (8)	0.0229 (8)	0.0065 (6)	−0.0041 (9)
Cl4	0.0514 (7)	0.1088 (11)	0.0296 (5)	−0.0003 (7)	−0.0031 (5)	−0.0046 (6)
Cl5	0.0560 (7)	0.0944 (10)	0.0324 (5)	−0.0004 (7)	0.0039 (5)	−0.0003 (6)
Cl6	0.0393 (6)	0.0929 (10)	0.0837 (9)	0.0038 (7)	0.0194 (6)	0.0158 (8)
Cl7	0.0457 (7)	0.1407 (15)	0.0755 (9)	0.0138 (8)	0.0286 (7)	0.0277 (9)
Cl8	0.0365 (6)	0.0757 (9)	0.0529 (6)	0.0033 (6)	−0.0062 (5)	0.0012 (6)
O1	0.0267 (13)	0.0592 (18)	0.0333 (14)	−0.0012 (13)	0.0027 (11)	−0.0040 (13)
O2	0.0325 (15)	0.061 (2)	0.0297 (14)	0.0029 (14)	−0.0006 (11)	−0.0028 (13)
O3	0.0297 (15)	0.0592 (19)	0.0321 (14)	0.0018 (13)	0.0018 (12)	−0.0064 (14)
O4	0.0253 (13)	0.0590 (16)	0.0382 (14)	0.0055 (12)	0.0033 (13)	0.0037 (16)
N1	0.0323 (18)	0.036 (2)	0.0333 (17)	−0.0036 (15)	−0.0036 (14)	0.0023 (15)
N2	0.0380 (19)	0.037 (2)	0.0248 (15)	−0.0019 (16)	−0.0042 (14)	0.0008 (14)
N3	0.0372 (19)	0.039 (2)	0.0306 (16)	0.0060 (16)	−0.0023 (15)	−0.0020 (15)
N4	0.0338 (18)	0.0336 (19)	0.0340 (17)	0.0033 (16)	−0.0009 (14)	−0.0018 (15)
C1	0.032 (2)	0.034 (2)	0.038 (2)	−0.0024 (17)	0.0021 (16)	0.0034 (17)
C2	0.0291 (17)	0.052 (2)	0.0345 (19)	−0.0034 (16)	−0.0023 (19)	−0.003 (2)
C3	0.029 (2)	0.057 (3)	0.045 (2)	0.000 (2)	−0.0037 (18)	0.000 (2)
C4	0.035 (2)	0.051 (3)	0.037 (2)	0.003 (2)	0.0070 (18)	−0.002 (2)
C5	0.037 (2)	0.048 (2)	0.0319 (19)	0.003 (2)	0.0021 (18)	−0.0021 (19)
C6	0.0289 (19)	0.035 (2)	0.0341 (19)	−0.0013 (17)	0.0013 (16)	0.0026 (18)
C7	0.035 (2)	0.048 (3)	0.0278 (19)	−0.003 (2)	0.0014 (17)	0.0032 (19)
C8	0.032 (2)	0.045 (3)	0.033 (2)	0.0002 (19)	−0.0099 (17)	0.0078 (19)
C9	0.0267 (19)	0.044 (2)	0.038 (2)	0.0003 (19)	−0.0029 (16)	0.0020 (19)
C10	0.035 (2)	0.048 (2)	0.0269 (18)	−0.0002 (19)	−0.0026 (16)	0.0021 (18)
C11	0.036 (2)	0.048 (3)	0.0275 (19)	−0.004 (2)	−0.0003 (16)	−0.0010 (18)
C12	0.032 (2)	0.048 (3)	0.0303 (19)	−0.0076 (19)	−0.0091 (17)	0.0041 (19)
C13	0.035 (2)	0.036 (2)	0.036 (2)	−0.0020 (18)	0.0018 (16)	0.0000 (18)
C14	0.037 (2)	0.064 (3)	0.045 (2)	0.004 (2)	0.0009 (19)	−0.003 (2)
C15	0.039 (2)	0.070 (3)	0.049 (3)	0.010 (2)	0.004 (2)	−0.006 (2)
C16	0.043 (3)	0.065 (3)	0.036 (2)	−0.002 (2)	0.0077 (19)	−0.006 (2)
C17	0.040 (2)	0.053 (3)	0.0287 (19)	−0.003 (2)	−0.0015 (17)	−0.0042 (19)
C18	0.041 (2)	0.039 (3)	0.033 (2)	−0.005 (2)	−0.0012 (18)	−0.0049 (18)
C19	0.037 (2)	0.030 (2)	0.035 (2)	0.0015 (18)	−0.0003 (17)	0.0061 (17)
C20	0.040 (2)	0.049 (3)	0.035 (2)	0.001 (2)	0.0070 (18)	0.003 (2)
C21	0.049 (3)	0.052 (3)	0.041 (2)	0.000 (2)	0.014 (2)	0.006 (2)
C22	0.035 (2)	0.048 (3)	0.059 (3)	−0.001 (2)	0.016 (2)	0.014 (2)
C23	0.036 (2)	0.050 (3)	0.047 (2)	−0.002 (2)	−0.001 (2)	0.004 (2)
C24	0.036 (2)	0.036 (2)	0.042 (2)	0.0010 (19)	0.0015 (19)	0.0034 (19)
C25	0.034 (2)	0.041 (3)	0.039 (2)	0.002 (2)	−0.0116 (17)	−0.0007 (18)
C26	0.035 (2)	0.048 (3)	0.0264 (19)	0.002 (2)	−0.0018 (16)	−0.0009 (18)
C27	0.038 (2)	0.042 (3)	0.032 (2)	−0.0019 (19)	0.0000 (16)	−0.0052 (18)
C28	0.034 (2)	0.046 (3)	0.039 (2)	−0.001 (2)	−0.0068 (17)	−0.002 (2)
C29	0.037 (2)	0.043 (2)	0.0307 (19)	−0.001 (2)	−0.0120 (17)	−0.0063 (19)
C30	0.040 (2)	0.048 (3)	0.034 (2)	0.002 (2)	0.0044 (18)	−0.001 (2)
C31	0.031 (2)	0.042 (2)	0.046 (2)	0.0037 (19)	0.0032 (18)	0.003 (2)
C32	0.043 (3)	0.062 (3)	0.044 (3)	0.008 (2)	0.006 (2)	0.008 (2)
C33	0.038 (2)	0.064 (3)	0.058 (3)	0.005 (2)	0.018 (2)	0.012 (3)
C34	0.032 (2)	0.054 (3)	0.060 (3)	−0.003 (2)	0.010 (2)	0.008 (2)
C35	0.032 (2)	0.040 (2)	0.049 (2)	0.0047 (18)	0.0009 (18)	0.007 (2)
C36	0.028 (2)	0.036 (2)	0.046 (2)	0.0055 (18)	0.0040 (17)	−0.0001 (18)

### Geometric parameters (Å, º)

**Table:**

Cu1—O1	1.888 (2)	C10—C11	1.513 (5)
Cu1—O4	1.898 (2)	C10—H10A	0.9700
Cu1—N4	2.000 (3)	C10—H10B	0.9700
Cu1—N1	2.001 (3)	C11—H11A	0.9700
Cu2—O2	1.898 (3)	C11—H11B	0.9700
Cu2—O3	1.913 (3)	C12—C13	1.443 (5)
Cu2—N2	1.995 (3)	C12—H12	0.9300
Cu2—N3	2.012 (3)	C13—C14	1.409 (5)
Cu2—O4^i^	2.854 (3)	C13—C18	1.415 (5)
Cl1—C2	1.744 (4)	C14—C15	1.369 (6)
Cl2—C4	1.741 (4)	C14—H14	0.9300
Cl3—C15	1.740 (4)	C15—C16	1.378 (6)
Cl4—C17	1.733 (4)	C16—C17	1.362 (6)
Cl5—C20	1.735 (4)	C16—H16	0.9300
Cl6—C22	1.733 (4)	C17—C18	1.437 (5)
Cl7—C33	1.748 (4)	C19—C24	1.405 (5)
Cl8—C35	1.730 (4)	C19—C20	1.422 (5)
O1—C1	1.292 (4)	C20—C21	1.369 (6)
O2—C18	1.283 (5)	C21—C22	1.395 (6)
O3—C19	1.298 (4)	C21—H21	0.9300
O4—C36	1.309 (4)	C22—C23	1.360 (6)
N1—C7	1.276 (5)	C23—C24	1.396 (5)
N1—C8	1.478 (5)	C23—H23	0.9300
N2—C12	1.291 (5)	C24—C25	1.447 (5)
N2—C11	1.473 (5)	C25—H25	0.9300
N3—C25	1.293 (5)	C26—C27	1.507 (5)
N3—C26	1.491 (5)	C26—H26A	0.9700
N4—C30	1.273 (5)	C26—H26B	0.9700
N4—C29	1.482 (5)	C27—C28	1.532 (5)
C1—C6	1.403 (5)	C27—H27A	0.9700
C1—C2	1.421 (5)	C27—H27B	0.9700
C2—C3	1.358 (5)	C28—C29	1.503 (6)
C3—C4	1.385 (5)	C28—H28A	0.9700
C3—H3	0.9300	C28—H28B	0.9700
C4—C5	1.365 (5)	C29—H29	0.9300
C5—C6	1.409 (5)	C30—C31	1.432 (6)
C5—H5	0.9300	C30—H30	0.9300
C6—C7	1.440 (5)	C31—C36	1.415 (6)
C7—H7	0.9300	C31—C32	1.417 (6)
C8—C9	1.513 (5)	C32—C33	1.356 (6)
C8—H8A	0.9700	C32—H32	0.9300
C8—H8B	0.9700	C33—C34	1.375 (6)
C9—C10	1.512 (5)	C34—C35	1.379 (5)
C9—H9A	0.9700	C34—H34	0.9300
C9—H9B	0.9700	C35—C36	1.414 (5)
			
O1—Cu1—O4	163.19 (11)	C14—C13—C12	116.8 (3)
O1—Cu1—N4	88.14 (12)	C18—C13—C12	121.6 (4)
O4—Cu1—N4	92.45 (12)	C15—C14—C13	119.9 (4)
O1—Cu1—N1	91.82 (11)	C15—C14—H14	120.0
O4—Cu1—N1	92.25 (12)	C13—C14—H14	120.0
N4—Cu1—N1	163.77 (13)	C14—C15—C16	120.5 (4)
O2—Cu2—O3	170.89 (13)	C14—C15—Cl3	120.4 (3)
O2—Cu2—N2	92.23 (12)	C16—C15—Cl3	119.1 (3)
O3—Cu2—N2	89.91 (12)	C17—C16—C15	120.4 (4)
O2—Cu2—N3	89.50 (12)	C17—C16—H16	119.8
O3—Cu2—N3	90.57 (12)	C15—C16—H16	119.8
N2—Cu2—N3	165.95 (14)	C16—C17—C18	122.6 (4)
O2—Cu2—O4^i^	84.56 (10)	C16—C17—Cl4	119.7 (3)
O3—Cu2—O4^i^	86.56 (10)	C18—C17—Cl4	117.7 (3)
N2—Cu2—O4^i^	90.47 (11)	O2—C18—C13	125.3 (4)
N3—Cu2—O4^i^	103.57 (11)	O2—C18—C17	119.8 (4)
C1—O1—Cu1	128.9 (2)	C13—C18—C17	115.0 (4)
C18—O2—Cu2	129.5 (2)	O3—C19—C24	124.2 (4)
C19—O3—Cu2	130.2 (2)	O3—C19—C20	120.2 (4)
C36—O4—Cu1	128.0 (3)	C24—C19—C20	115.5 (4)
C7—N1—C8	116.8 (3)	C21—C20—C19	122.5 (4)
C7—N1—Cu1	123.2 (3)	C21—C20—Cl5	119.2 (3)
C8—N1—Cu1	120.0 (3)	C19—C20—Cl5	118.3 (3)
C12—N2—C11	115.8 (3)	C20—C21—C22	119.8 (4)
C12—N2—Cu2	124.3 (3)	C20—C21—H21	120.1
C11—N2—Cu2	119.9 (2)	C22—C21—H21	120.1
C25—N3—C26	115.0 (3)	C23—C22—C21	119.8 (4)
C25—N3—Cu2	124.5 (3)	C23—C22—Cl6	120.5 (4)
C26—N3—Cu2	120.5 (2)	C21—C22—Cl6	119.7 (3)
C30—N4—C29	115.8 (3)	C22—C23—C24	120.7 (4)
C30—N4—Cu1	123.6 (3)	C22—C23—H23	119.6
C29—N4—Cu1	120.3 (2)	C24—C23—H23	119.6
O1—C1—C6	124.5 (3)	C23—C24—C19	121.6 (4)
O1—C1—C2	119.7 (3)	C23—C24—C25	116.5 (4)
C6—C1—C2	115.8 (3)	C19—C24—C25	121.6 (4)
C3—C2—C1	123.3 (4)	N3—C25—C24	127.3 (4)
C3—C2—Cl1	119.5 (3)	N3—C25—H25	116.4
C1—C2—Cl1	117.2 (3)	C24—C25—H25	116.4
C2—C3—C4	119.3 (4)	N3—C26—C27	112.2 (3)
C2—C3—H3	120.4	N3—C26—H26A	109.2
C4—C3—H3	120.4	C27—C26—H26A	109.2
C5—C4—C3	120.5 (4)	N3—C26—H26B	109.2
C5—C4—Cl2	120.3 (3)	C27—C26—H26B	109.2
C3—C4—Cl2	119.2 (3)	H26A—C26—H26B	107.9
C4—C5—C6	120.3 (4)	C26—C27—C28	115.4 (3)
C4—C5—H5	119.8	C26—C27—H27A	108.4
C6—C5—H5	119.8	C28—C27—H27A	108.4
C1—C6—C5	120.7 (4)	C26—C27—H27B	108.4
C1—C6—C7	121.6 (3)	C28—C27—H27B	108.4
C5—C6—C7	117.5 (4)	H27A—C27—H27B	107.5
N1—C7—C6	127.6 (4)	C29—C28—C27	113.5 (3)
N1—C7—H7	116.2	C29—C28—H28A	108.9
C6—C7—H7	116.2	C27—C28—H28A	108.9
N1—C8—C9	111.9 (3)	C29—C28—H28B	108.9
N1—C8—H8A	109.2	C27—C28—H28B	108.9
C9—C8—H8A	109.2	H28A—C28—H28B	107.7
N1—C8—H8B	109.2	N4—C29—C28	110.7 (3)
C9—C8—H8B	109.2	N4—C29—H29	124.6
H8A—C8—H8B	107.9	C28—C29—H29	124.6
C10—C9—C8	114.3 (3)	N4—C30—C31	127.4 (4)
C10—C9—H9A	108.7	N4—C30—H30	116.3
C8—C9—H9A	108.7	C31—C30—H30	116.3
C10—C9—H9B	108.7	C36—C31—C32	120.3 (4)
C8—C9—H9B	108.7	C36—C31—C30	122.9 (4)
H9A—C9—H9B	107.6	C32—C31—C30	116.9 (4)
C9—C10—C11	113.3 (3)	C33—C32—C31	120.2 (4)
C9—C10—H10A	108.9	C33—C32—H32	119.9
C11—C10—H10A	108.9	C31—C32—H32	119.9
C9—C10—H10B	108.9	C32—C33—C34	121.3 (4)
C11—C10—H10B	108.9	C32—C33—Cl7	118.7 (4)
H10A—C10—H10B	107.7	C34—C33—Cl7	120.0 (4)
N2—C11—C10	110.3 (3)	C33—C34—C35	119.4 (4)
N2—C11—H11A	109.6	C33—C34—H34	120.3
C10—C11—H11A	109.6	C35—C34—H34	120.3
N2—C11—H11B	109.6	C34—C35—C36	122.4 (4)
C10—C11—H11B	109.6	C34—C35—Cl8	119.0 (3)
H11A—C11—H11B	108.1	C36—C35—Cl8	118.6 (3)
N2—C12—C13	127.1 (4)	O4—C36—C35	120.4 (4)
N2—C12—H12	116.4	O4—C36—C31	123.3 (4)
C13—C12—H12	116.4	C35—C36—C31	116.4 (3)
C14—C13—C18	121.6 (4)		
			
O4—Cu1—O1—C1	120.7 (4)	N2—C12—C13—C14	179.2 (4)
N4—Cu1—O1—C1	−146.9 (3)	N2—C12—C13—C18	0.2 (7)
N1—Cu1—O1—C1	16.8 (3)	C18—C13—C14—C15	1.2 (7)
N2—Cu2—O2—C18	−2.0 (4)	C12—C13—C14—C15	−177.9 (4)
N3—Cu2—O2—C18	−168.1 (4)	C13—C14—C15—C16	−1.3 (8)
N2—Cu2—O3—C19	−151.8 (3)	C13—C14—C15—Cl3	179.5 (3)
N3—Cu2—O3—C19	14.2 (3)	C14—C15—C16—C17	0.5 (8)
O1—Cu1—O4—C36	108.6 (5)	Cl3—C15—C16—C17	179.7 (4)
N4—Cu1—O4—C36	16.9 (3)	C15—C16—C17—C18	0.5 (7)
N1—Cu1—O4—C36	−147.6 (3)	C15—C16—C17—Cl4	−179.1 (4)
O1—Cu1—N1—C7	−12.0 (3)	Cu2—O2—C18—C13	2.5 (6)
O4—Cu1—N1—C7	−175.7 (3)	Cu2—O2—C18—C17	−177.6 (3)
N4—Cu1—N1—C7	77.6 (6)	C14—C13—C18—O2	179.7 (4)
O1—Cu1—N1—C8	166.3 (3)	C12—C13—C18—O2	−1.3 (7)
O4—Cu1—N1—C8	2.6 (3)	C14—C13—C18—C17	−0.1 (6)
N4—Cu1—N1—C8	−104.2 (5)	C12—C13—C18—C17	178.8 (4)
O2—Cu2—N2—C12	0.9 (3)	C16—C17—C18—O2	179.4 (4)
O3—Cu2—N2—C12	−170.2 (4)	Cl4—C17—C18—O2	−1.0 (6)
N3—Cu2—N2—C12	97.8 (6)	C16—C17—C18—C13	−0.7 (6)
O2—Cu2—N2—C11	−176.9 (3)	Cl4—C17—C18—C13	178.9 (3)
O3—Cu2—N2—C11	12.0 (3)	Cu2—O3—C19—C24	−11.7 (6)
N3—Cu2—N2—C11	−80.0 (6)	Cu2—O3—C19—C20	170.4 (3)
O2—Cu2—N3—C25	−179.5 (3)	O3—C19—C20—C21	174.3 (4)
O3—Cu2—N3—C25	−8.6 (3)	C24—C19—C20—C21	−3.8 (6)
N2—Cu2—N3—C25	83.3 (7)	O3—C19—C20—Cl5	−3.5 (5)
O2—Cu2—N3—C26	2.2 (3)	C24—C19—C20—Cl5	178.4 (3)
O3—Cu2—N3—C26	173.1 (3)	C19—C20—C21—C22	1.3 (7)
N2—Cu2—N3—C26	−95.0 (6)	Cl5—C20—C21—C22	179.2 (3)
O1—Cu1—N4—C30	−169.2 (4)	C20—C21—C22—C23	1.4 (7)
O4—Cu1—N4—C30	−6.0 (4)	C20—C21—C22—Cl6	−176.8 (3)
N1—Cu1—N4—C30	100.7 (5)	C21—C22—C23—C24	−1.5 (7)
O1—Cu1—N4—C29	16.0 (3)	Cl6—C22—C23—C24	176.7 (3)
O4—Cu1—N4—C29	179.2 (3)	C22—C23—C24—C19	−1.2 (6)
N1—Cu1—N4—C29	−74.2 (6)	C22—C23—C24—C25	−175.2 (4)
O4^i^—Cu2—O2—C18	88.2 (4)	O3—C19—C24—C23	−174.4 (4)
O4^i^—Cu2—O3—C19	117.7 (3)	C20—C19—C24—C23	3.7 (6)
O4^i^—Cu2—N2—C12	−83.7 (3)	O3—C19—C24—C25	−0.7 (7)
O4^i^—Cu2—N2—C11	98.5 (3)	C20—C19—C24—C25	177.3 (4)
O4^i^—Cu2—N3—C25	−95.2 (3)	C26—N3—C25—C24	179.6 (4)
O4^i^—Cu2—N3—C26	86.5 (3)	Cu2—N3—C25—C24	1.3 (7)
Cu1—O1—C1—C6	−11.3 (5)	C23—C24—C25—N3	179.7 (4)
Cu1—O1—C1—C2	171.3 (3)	C19—C24—C25—N3	5.7 (8)
O1—C1—C2—C3	179.2 (4)	C25—N3—C26—C27	−102.5 (4)
C6—C1—C2—C3	1.6 (6)	Cu2—N3—C26—C27	75.9 (4)
O1—C1—C2—Cl1	0.1 (5)	N3—C26—C27—C28	64.9 (4)
C6—C1—C2—Cl1	−177.5 (3)	C26—C27—C28—C29	−173.0 (3)
C1—C2—C3—C4	−0.9 (7)	C30—N4—C29—C28	−105.5 (4)
Cl1—C2—C3—C4	178.2 (3)	Cu1—N4—C29—C28	69.7 (4)
C2—C3—C4—C5	0.3 (7)	C27—C28—C29—N4	58.2 (4)
C2—C3—C4—Cl2	−178.3 (3)	C29—N4—C30—C31	171.4 (4)
C3—C4—C5—C6	−0.4 (6)	Cu1—N4—C30—C31	−3.6 (7)
Cl2—C4—C5—C6	178.2 (3)	N4—C30—C31—C36	7.0 (7)
O1—C1—C6—C5	−179.2 (4)	N4—C30—C31—C32	−171.1 (4)
C2—C1—C6—C5	−1.7 (5)	C36—C31—C32—C33	−1.6 (7)
O1—C1—C6—C7	−4.4 (6)	C30—C31—C32—C33	176.5 (4)
C2—C1—C6—C7	173.0 (3)	C31—C32—C33—C34	−0.6 (7)
C4—C5—C6—C1	1.2 (6)	C31—C32—C33—Cl7	179.7 (4)
C4—C5—C6—C7	−173.8 (4)	C32—C33—C34—C35	1.9 (7)
C8—N1—C7—C6	−175.9 (4)	Cl7—C33—C34—C35	−178.4 (4)
Cu1—N1—C7—C6	2.4 (6)	C33—C34—C35—C36	−1.0 (7)
C1—C6—C7—N1	8.7 (7)	C33—C34—C35—Cl8	178.5 (4)
C5—C6—C7—N1	−176.4 (4)	Cu1—O4—C36—C35	161.8 (3)
C7—N1—C8—C9	−103.0 (4)	Cu1—O4—C36—C31	−18.3 (6)
Cu1—N1—C8—C9	78.6 (4)	C34—C35—C36—O4	178.8 (4)
N1—C8—C9—C10	63.3 (4)	Cl8—C35—C36—O4	−0.7 (5)
C8—C9—C10—C11	−172.3 (3)	C34—C35—C36—C31	−1.1 (6)
C12—N2—C11—C10	−109.1 (4)	Cl8—C35—C36—C31	179.3 (3)
Cu2—N2—C11—C10	68.9 (4)	C32—C31—C36—O4	−177.5 (4)
C9—C10—C11—N2	60.6 (4)	C30—C31—C36—O4	4.4 (7)
C11—N2—C12—C13	177.6 (4)	C32—C31—C36—C35	2.4 (6)
Cu2—N2—C12—C13	−0.3 (6)	C30—C31—C36—C35	−175.6 (4)

Symmetry code: (i) *x*, *y*+1, *z*.

### Hydrogen-bond geometry (Å, º)

**Table:**

*D*—H···*A*	*D*—H	H···*A*	*D*···*A*	*D*—H···*A*
C10—H10*B*···O3	0.97	2.46	3.073 (5)	121
C27—H27*A*···O2	0.97	2.50	3.098 (5)	120
C28—H28*A*···O1	0.97	2.57	3.137 (5)	118
